# Supplementary material for: Active site specificity profiling datasets of matrix metalloproteinases (MMPs) 1, 2, 3, 7, 8, 9, 12, 13 and 14
Source: Data Brief. 2016 Feb 22;7:299–310. doi: 10.1016/j.dib.2016.02.036 (PMC4777984; doi:10.1016/j.dib.2016.02.036)
Supplement: Supplementary file 10 — Supplementary material [file mmc10.zip › WebPICS_hMMP13_T_1%/P2.html]

 

PICS results


|  |  |
| --- | --- |
| **P2\_A**  27 in 143 sites   18.9 %    effects > 10 perc. pnts.  (vice-versa in brackets)  P1\_G: 18.8 (19.6)   P1prime\_W: 15.9 (47.8)   P2prime\_K: 12.4 (24.0) |  |
| **P2\_F**  8 in 143 sites   5.6 %    effects > 10 perc. pnts.  (vice-versa in brackets)  P1\_P: 27.7 (15.8)   P1prime\_V: 38.8 (19.4)   P3prime\_H: 17.3 (12.6)   P3prime\_N: 19.4 (19.4) |  |
| **P2\_H**  4 in 143 sites   2.8 %    effects > 10 perc. pnts.  (vice-versa in brackets)  P1\_P: 40.2 (11.5)   P1\_W: 23.6 (47.2)   P1prime\_Q: 43.7 (19.4)   P2prime\_Q: 41.6 (13.9)   P3prime\_H: 42.3 (15.4) |  |
| **P2\_M**  4 in 143 sites   2.8 %    effects > 10 perc. pnts.  (vice-versa in brackets)  P2prime\_M: 47.2 (47.2) |  |
| **P2\_Q**  8 in 143 sites   5.6 %    effects > 10 perc. pnts.  (vice-versa in brackets)  P1prime\_M: 20.8 (27.7)   P3prime\_S: 28.4 (17.5) |  |
| **P2\_S**  19 in 143 sites   13.3 %    effects > 10 perc. pnts.  (vice-versa in brackets)  P1\_S: 22.5 (32.9)   P2prime\_V: 11.6 (10.5) |  |
| **P2\_V**  13 in 143 sites   9.1 %    effects > 10 perc. pnts.  (vice-versa in brackets)  P3prime\_A: 25.9 (11.6) |  |
